# *Enterococcus hirae* bacteremia associated with acute pyelonephritis in a patient with alcoholic cirrhosis: a case report and literature review

**DOI:** 10.1186/s12879-021-06707-2

**Published:** 2021-09-23

**Authors:** Tomoaki Nakamura, Kazuhiro Ishikawa, Takahiro Matsuo, Fujimi Kawai, Yuki Uehara, Nobuyoshi Mori

**Affiliations:** 1 Department of Infectious Diseases, St. Luke’s International Hospital, 9-1, Akashi-cho, Chuo-ku, Tokyo, Japan; 2 St. Luke’s International University Library, Tokyo, Japan; 3 Department of Clinical Laboratory, St. Luke’s International Hospital, Tokyo, Japan; 4 Department of Microbiology, Faculty of Medicine, Juntendo University, Tokyo, Japan; 5 Department of General Medicine, Faculty of Medicine, Juntendo University, Tokyo, Japan

**Keywords:** *Enterococcus hirae*, Urinary tract infection, Alcoholic cirrhosis, Case report

## Abstract

**Background:**

Infections caused by *Enterococcus hirae* are common in animals, with instances of transmission to humans being rare. Further, few cases have been reported in humans because of the difficulty in identifying the bacteria. Herein, we report a case of pyelonephritis caused by *E. hirae* bacteremia and conduct a literature review on *E. hirae* bacteremia.

**Case presentation:**

A 57-year-old male patient with alcoholic cirrhosis and neurogenic bladder presented with fever and chills that had persisted for 3 days. Physical examination revealed tenderness of the right costovertebral angle. Matrix-assisted laser desorption ionization-time of flight mass spectrometry (MALDI-TOF MS) of the patient’s blood and urine samples revealed the presence of *E. hirae*, and pyelonephritis was diagnosed. The patient was treated successfully with intravenous ampicillin followed by oral linezolid for a total of three weeks.

**Conclusion:**

The literature review we conducted revealed that *E. hirae* bacteremia is frequently reported in urinary tract infections, biliary tract infections, and infective endocarditis and is more likely to occur in patients with diabetes, liver cirrhosis, and chronic kidney disease. However, mortality is not common because of the high antimicrobial susceptibility of *E. hirae*. With the advancements in MALDI-TOF MS, the number of reports of *E. hirae* infections has also increased, and clinicians need to consider *E. hirae* as a possible causative pathogen of urinary tract infections in patients with known risk factors.

## Background

*Enterococcus hirae* primarily causes zoonosis [1, 2], with human infections being relatively rare. Nevertheless, pyelonephritis [3–5], infective endocarditis [6–11], and biliary tract infections [5, 12] due to *E. hirae* have been reported in human patients. Although *E. hirae* has been found to cause these severe diseases in humans, few cases have been reported because of the difficulty in identifying the bacteria, and the lack of comprehensive reports on clinical characteristics and treatments [3].

Matrix-assisted laser desorption ionization-time of flight mass spectrometry (MALDI-TOF MS) has recently emerged as an important diagnostic tool, characterized by its high speed, ease of use, and low per sample cost compared to those of conventional diagnostic tools [13]. Therefore, greater progress in the analysis of a variety of bacterial species that have been difficult to identify in the past is expected [13]. In a case of urinary tract infection, *E. hirae* was rapidly and correctly identified using MALDI-TOF MS, without any complementary tests [14]. Here, we report a case of bacteremia secondary to pyelonephritis caused by *E. hirae* identified by MALDI-TOF MS, which was successfully treated with ampicillin followed by linezolid. Furthermore, we conducted a literature review on bacteremia caused by *E. hirae*.

## Case presentation

**Fig. 1:**
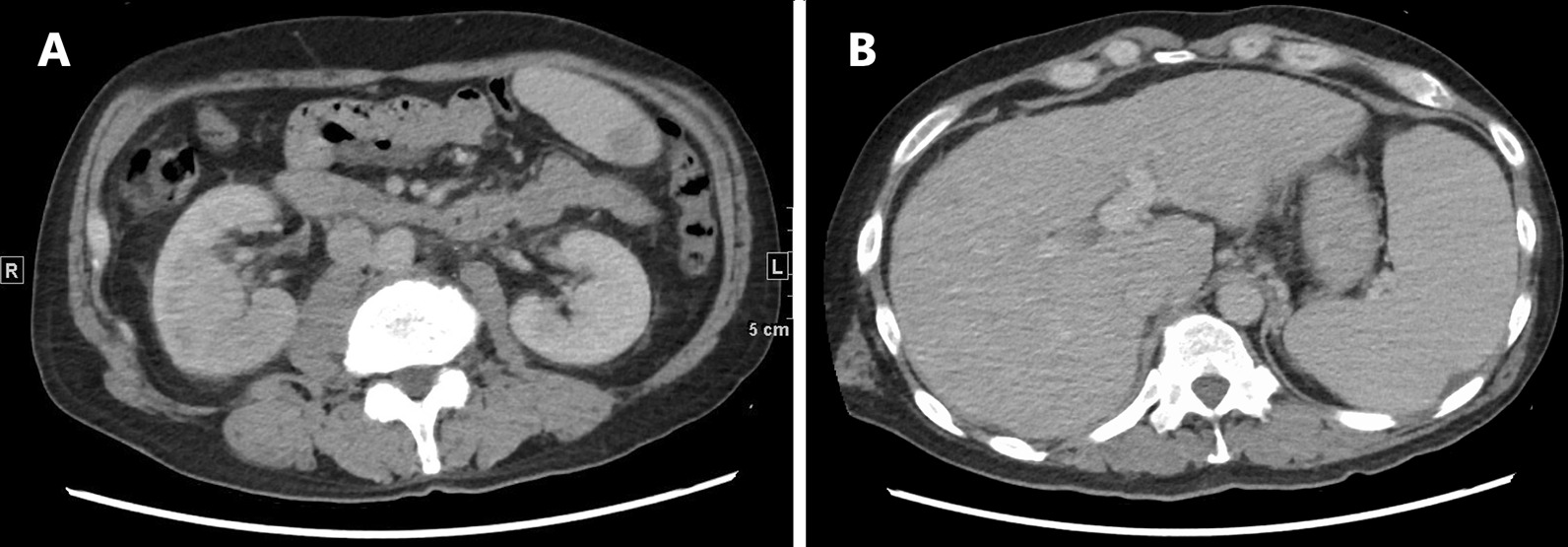
Contrast-enhanced computed tomographic images revealing heterogeneous enhancement of both kidneys in **A**, and a liver with a blunt edge and irregular surface in **B**

A 57-year-old male with a history of neurogenic bladder caused by cerebral palsy presented to our emergency department with fever and chills that had persisted for 3 days. He had a history of alcoholic cirrhosis classified as Child–Pugh class C treated with rifaximin, lactulose, and branched-chain amino acid supplementation. The patient reported daily consumption of 500 mL of Shochu (a traditional Japanese distilled spirit). He had no allergies or significant family history. He was unemployed and denied any recent contact with animals. The patient was diagnosed with a urinary tract infection at a nearby clinic and was prescribed oral cefcapene 2 days before admission. The patient was conscious on admission with a Glasgow Coma Scale of E4V5M6, body temperature of 36.9 °C, blood pressure of 104/52 mmHg, pulse rate of 82/min, respiratory rate of 20/min, and oxygen saturation of 95% on room air. On physical examination, tenderness of the right costovertebral angle was noted. Laboratory findings revealed a normal white blood cell (WBC) count of 6,000 /μL, hemoglobin level of 12.3 g/dL, platelet count of 48,000 /μL, creatinine level of 0.92 mg/dL, serum albumin level of 2.9 g/dL, total bilirubin level of 2.7 mg/dL, and C-reactive protein level of 13 mg/dL. Urinalysis showed protein 2 + , occult blood 2 + , and WBC 2 + . Urine Gram staining revealed gram-positive chains with phagocytosis. Contrast-enhanced computed tomography of the abdomen revealed mild swelling of the kidneys, increased surrounding fat tissue density, and a dull edge and uneven surface of the liver (Fig. 1). We first administered 1 g of intravenous (IV) ceftriaxone every 24 h. On day 2, we added 2 g of IV ampicillin every 4 h because streptococci were cultured from blood and urine samples obtained on admission (BacT/ALERT FA Plus, BacT/ALERT 3D [bioMérieux Inc.]). On day 4, a transthoracic echocardiogram revealed no evidence of infective endocarditis. On day 5, final culture results revealed *E. hirae* by matrix-assisted laser desorption ionization-time of flight mass spectrometry (MALDI-TOF MS) (MALDI Biotyper [Bruker Daltonics]) and VITEK2 Compact (bioMérieux Inc.). The minimum inhibitory concentrations measured by MicroScan WalkAway 96 Plus and PC1J panel(Beckman Coulter Inc.) for this strain were as follows: penicillin G 0.25 μg/mL, ampicillin 0.25 μg/mL, vancomycin 1 μg/mL, levofloxacin ≤ 0.5 μg/mL, teicoplanin ≤ 2 μg/mL, and linezolid 2 μg/mL (Table 1). We switched to ampicillin IV (2 g every 6 h). Blood cultures performed on day 5 were negative. Because his low-grade fever persisted, we switched to oral linezolid 600 mg every 12 h on day 11, considering possible drug fever. Thereafter, the patient defervesced and was discharged on day 15. He completed a course of oral linezolid for 3 weeks in total, and his condition resolved without any relapse of symptoms at the 10-month follow-up.

**Table 1:** Antimicrobial susceptibility of the *Enterococcus hirae* isolated from blood culture in this case

Antimicrobials	MIC (μg/mL)	Susceptibility^a^
Penicillin G	0.25	N/A
Ampicillin	0.25	Susceptible
Vancomycin	1	Susceptible
Levofloxacin	≤ 0.5	Susceptible
Teicoplanin	≤ 2	Susceptible
Linezolid	2	Susceptible

*MIC* Minimal inhibitory concentration

^a^Based on the European Committee on Antimicrobial Susceptibility Testing (EUCAST) Clinical Breakpoints v.11.0, for *Enterococcus* spp.[15]

### Methods of literature review

**Fig. 2:**
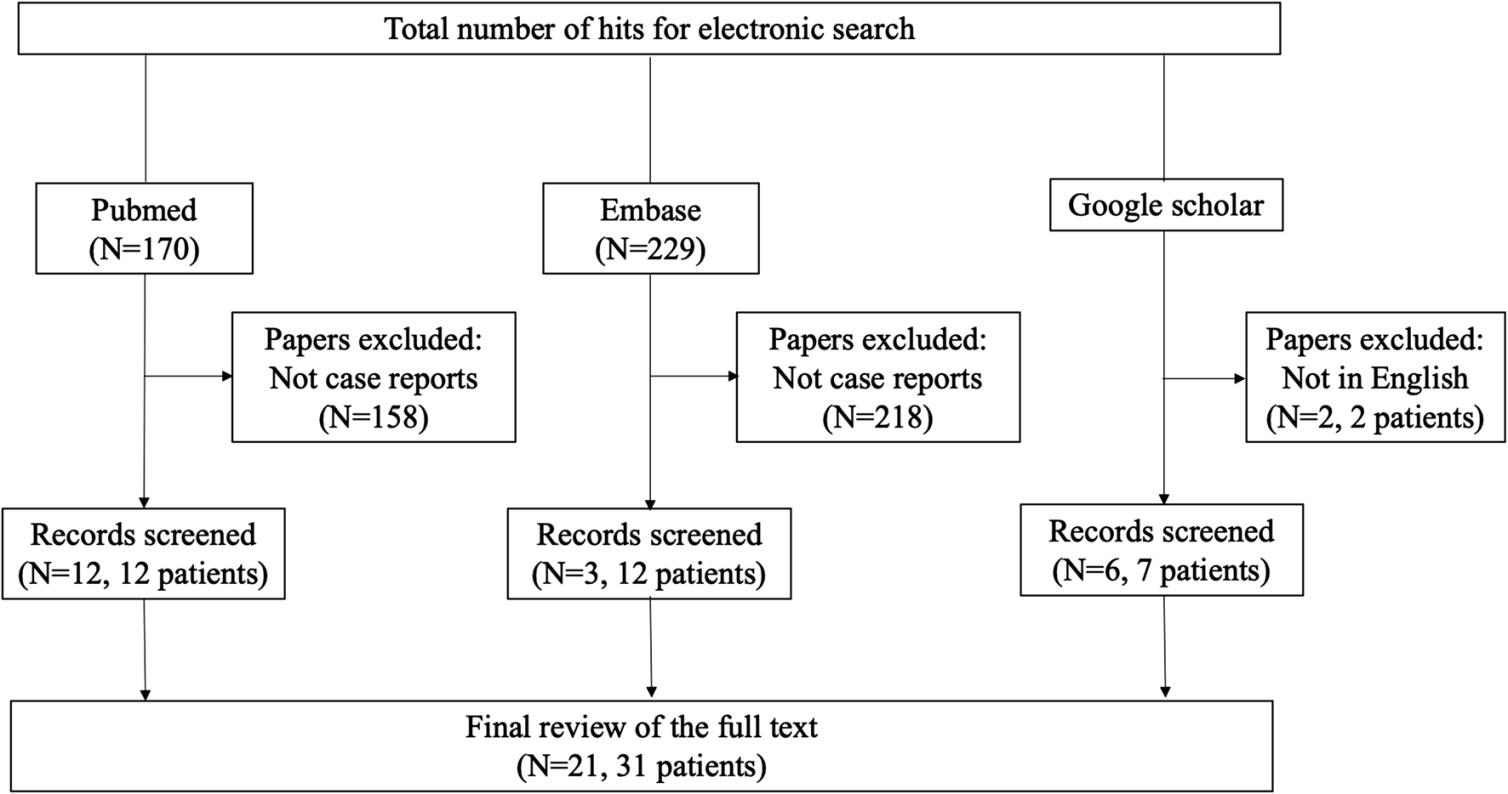
Literature review flow chart

Two authors independently reviewed the titles and abstracts of database records, retrieved full texts for eligibility assessment, and extracted data from these case reports. We ran searches on the PubMed database (up to May 2020) using the keywords ((("Enterococcus hirae"[Mesh]) OR ("Enterococcus hirae"[TW]) OR (hirae[TIAB])) AND ((Bacteremia[MH]) OR (bacteremia*[TIAB] OR bacteraemia*[TIAB]))) OR ((("Enterococcus hirae"[Mesh]) OR ("Enterococcus hirae"[TW]) OR (hirae[TIAB])) AND Humans[MH]), and the Embase database using the keywords (('bacteremia'/exp OR 'gram negative sepsis'/exp OR bacteraemia* OR bacteremia*) AND ('enterococcus hirae'/exp OR hirae)) OR (('enterococcus hirae'/exp OR hirae) AND [humans]/lim). PubMed and Embase searches generated 170 and 229 articles, respectively. Of these, 158 and 218 articles from PubMed and Embase, respectively, were excluded because they were not case reports (Fig. 2). We searched Google Scholar and identified eight more human cases. Manuscripts not written in English were excluded. Finally, we reviewed 21 articles that included 31 strains from human sources.

## Discussion and conclusion

*Enterococcus hirae* was first identified by Farrow et al. in 1985 [16]. It has been reported that although animal species such as chickens, rats, birds, and cats are commonly found to be infected [1, 2], human infections are relatively rare [17]. Only 31 human cases of *E. hirae* have been reported (Table 2). Of these, urinary tract infections [3–5, 12, 14, 18], biliary tract infections [5, 12], and infective endocarditis [6–11] accounted for the majority of cases, with catheter-related bloodstream infections [12, 19], peritonitis [20, 21], splenic abscess [22], and pneumonia [17] also being reported. Patients were predominantly male (n = 20, 64.5%), similar to predominance in infections caused by other *Enterococcus* spp. [23], Furthermore, no age trend was observed (median: 63 years) [23]. The common underlying diseases were diabetes mellitus (n = 12, 39%), liver cirrhosis (n = 4, 13%), and chronic kidney disease (n = 4, 13%). Occurrence of diabetes mellitus and liver cirrhosis was consistent with previous reports of *Enterococcus* spp. Malignant tumors were found to be less common [23]. This case of a middle-aged male with underlying alcoholic cirrhosis and chronic kidney disease was consistent with the trend uncovered in the literature review.

**Table 2:** Summary of the previously reported human cases with *Enterococcus hirae*

Case	References	Age	Gender	Year	Underlying diseases	Chief complaint	Method of *E. hirae* identification	Diagnosis	treatment	Outcome
1	Gilad et al. [24]	49	Male	1998	ESRD with hemodialysis, Indwelling central venous catheter	Fever	Rapid ID 32 Strep system	Septicemia	VCM + TOB	Complete resolution
2	Tan et al. [12]	82	Female	2000	DM	N/A	6.5% NaCl tolerance and growth on bile-esculin agar with esculin hydrolysis	Urinary tract infection	AMPC + GEM	Complete resolution
3	Tan et al. [12]	80	Male	2001	Biliary tract disease	N/A	6.5% NaCl tolerance and growth on bile-esculin agar with esculin hydrolysis	Biliary tract infection	CMZ + Operation	Complete resolution
4	Poyart et al. [6]	72	Male	2002	Coronary artery disease	Fever, Chills, Progressive malaise, Generalized weakness	(sodA gene) sequencing	Native valve Endocarditis	ABPC + GM 4 weeks, RFP 3 weeks → ABPC + GM po Readmission: VCM + GM 6 weeks → ABPC po total 8 weeks	Complete resolution
5	Tan et al. [12]	50	Male	2002	ESRD	N/A	6.5% NaCl tolerance and growth on bile-esculin agar with esculin hydrolysis	Primary bacteremia	VCM	Complete resolution
6	Tan et al. [12]	55	Male	2003	N/A	N/A	6.5% NaCl tolerance and growth on bile-esculin agar with esculin hydrolysis	Urinary tract infection	ABPC	Complete resolution
7	Tan et al. [12]	63	Male	2004	Biliary tract disease	N/A	6.5% NaCl tolerance and growth on bile-esculin agar with esculin hydrolysis	Biliary tract infection	ABPC/SBT + biliary drainage	Complete resolution
8	Tan et al. [12]	69	Female	2004	N/A	N/A	6.5% NaCl tolerance and growth on bile-esculin agar with esculin hydrolysis	Urinary tract infection	ABPC/SBT	Complete resolution
9	Tan et al. [12]	57	Male	2006	Tongue cancer	N/A	6.5% NaCl tolerance and growth on bile-esculin agar with esculin hydrolysis	Catheter Associate infection	VCM + Removal of catheter	Complete resolution
10	Vinh et al. [9]	80	Male	2006	DM, Hypercholesterolemia, Coronary artery disease, Resection of malignant colonic polyp	Dyspnea, Vague epigastric discomfort	VITEK 2 automated system (bioMériux)	Native-valve bacterial endocarditis	ABPC 6 weeks after aortic replacement	Complete resolution
11	Tan et al. [12]	59	Male	2008	Pancreatic cancer with obstructive jaundice	N/A	6.5% NaCl tolerance and growth on bile-esculin agar with esculin hydrolysis	Biliary tract infection	VCM + IPM + Surgical intervention	Died
12	Tan et al. [12]	69	Female	2008	Lung cancer on chemotherapy	N/A	6.5% NaCl tolerance and growth on bile-esculin agar with esculin hydrolysis	CatheterAssociate infection	VCM + Removal of catheter	Complete resolution
13	Canalejo et al. [25]	55	Male	2008	DM	Low back pain, Fever, Chills	VITEK 2 automated system (bioMériux), rRNA gene sequencing	Spondylodiscitis	ABPC + GM 8 Weeks → Surgery → LVFX po + ST 6 months	Complete resolution
14	Nicolosi et al. [26]	63	Male	2009	N/A	N/A	Unknown	Bacteremia	N/A	N/A
15	Chan et al. [5]	62	Female	2010	N/A	Fever, Chills, Urinary irritation	BD Phoenix ID/AST Panel Inoculation System	Acute pyelonephritis	CEZ + GM → ABPC → AMPC for total 12 days	Complete resolution
16	Chan et al. [5]	86	Female	2010	Congestive heart failure, HT, Valvular heart disease, Parkinsonism, Dementia, Recent history of hospitalization	Hypotensive, Febrile, Tachycardiac, Tachypneic	BD Phoenix ID/AST Panel Inoculation System	Acute cholangitis	CMZ 16 days → oral antibiotics (unknown) total 23 days	Complete resolution
17	Talarmin et al. [7]	78	Female	2011	DM, HT, Aortic valve replacement with a bioprosthetic valve	Fever, Generalized weakness, Weight loss	I6S rRNA sequencing	Prosthetic valve endocarditis	AMPC + GM 2 weeks → AMPC + RFP 4 weeks → relapse 4 months after discontinuation of antibiotics therapy, same antibiotics started as for the initial episode for total 6 weeks	Relapse → Complete resolution
18	Sim et al. [20]	61	Male	2012	Alcoholic liver cirrhosis, DM	Abdominal pain, Fever, Chills, Generalized weakness	Automated MicroScan WalkAway system; sugar fermentation tests	Spontaneous bacterial peritonitis	CTX → VCM + CPFX → ABPC total 17 days	Complete resolution
19	Brulé et al. [18]	44	Male	2013	Alcoholic liver disease, Atrial fibrillation, Dilated cardiomyopathy	Fever, Diarrhea, Vomit	Gel electrophoresis	Bacteremia, Pyelonephritis	CTRX + MNZ → add AMK → nephrectomy → AMPC total 21 days	Complete resolution
20	Anghinah R et al. [10]	56	Male	2013	HT, DM, Hypercholesterolemia, Cardiac arrhythmia with surgical ablation, Surgical removal of a gastric leiomyoma	Slurred speech, Weight loss, Generalized fatigue, Depressive symptoms, Fever	Unknown	Native valve endocarditis	Oxacillin + GM → ABPC → ABPC + RFP total 4 weeks + replacement of the aortic valve → RFP + AMPC 2 weeks	Complete resolution
21	Alfouzan et al.[22]	48	Female	2014	DM	Abdominal pain, Productive cough, Fever	BD Phoenix Automated Microbiology System and DNA sequencing	Multiple splenic abscesses	PIPC/TAZ + VCM + MNZ → Splenectomy → PIPC/TAZ + ABPC + LZD total 2 weeks	Complete resolution
22	Dicpinigaitis et al. [27]	85	Female	2015	HT, Hyperlipidemia	Nausea, Vomit, Abdominal pain	MALDI-TOF MS	Acute Pancreatitis	PIPC/TAZ → CFPM → ABPC total 14 days	Complete resolution
23	Bourafa et a. [14]	50	Male	2015	BPH, DM, Urinary catheterization	Dysuria with cloudy urine, Suprapubic pain, Urinary frequency, and urgency	MALDI-TOF MS	Symptomatic lower UTI	APBC + GM total 10 days	Complete resolution
24	Paosinho et al. [3]	78	Female	2016	Atrial fibrillation, Chronic renal disease	Nausea, Lipothymia, Generalized weakness	Unknown	Acute pyelonephritis	AMPC/CVA → PIPC/TAZ total 14 days	Complete resolution
25	Atas et al. [21]	70	Female	2017	CKD, Dialysis	Abdominal pain, Cloudy dialysate	Unknown	Peritonitis	Intraperitoneal CXM-AX + CPFX PO → did not respond to therapy → VCM 3 weeks → discharge → relapse → Intraperitoneal VCM 3 weeks	Relapse → Complete resolution
26	Hee Lee et al. [4]	78	Male	2017	DM, HT, Coronary arterial occlusive disease	Left flank pain, Febrile sensation	BacT/ALERT 3D Microbial Detection System (bioMérieux Inc., Durham)	Acute Pyelonephritis	CTRX → CPFX po 14 days	Complete resolution
27	Hee Lee et al. [4]	74	Male	2017	DM, HT, Coronary arterial occlusive disease	Left flank pain, Febrile sensation, Chills	BacT/ALERT 3D Microbial Detection System (bioMérieux Inc., Durham)	Acute pyelonephritis	CTRX → CPFX po 14 days	Complete resolution
28	Gittemeier et al. [8]	70	Male	2019	N/A	Bilateral leg edema, Dyspnea on exertion, Fatigue	MALDI-TOF MS	Aortic valve endocarditis	VCM → ABPC + CTRX → Aortic valve replacement → CTRX + PCG total 6 weeks	Complete resolution
29	Merlo et al. [17]	57	Male	2019	DM, COPD, Hepatic cirrhosis Child–Pugh B secondary to HCV	Dyspnea, Disorientation, Fever	MALDI-TOF MS	Pneumonia	PIPC/TAZ + AZM + Rifaximine PO → AMPC/CVA total of 8 days	Complete resolution
30	Pinkes et al. [11]	67	Female	2019	COPD, Recurrent DVT, Atrial fibrillation, HT, Hypothyroidism, Hodgkin’s lymphoma	fever, hypotension, atrial fibrillation with a rapid ventricular response, and a two-week history of lightheadedness	MALDI-TOF–MS	Native-valve endocarditis	Aortic valve replacement, ABPC + CTRX total of 6 weeks	Complete resolution
31	Brayer et al. [19]	7 months	Male	2019	Gastroschisis, Jejunal atresia	Fussiness, Fever	Vitek 2 system (bioM.rieux)	Catheter associated infection	VCM + PIPC/TAZ → VCM → VCM + CTRX → ABPC + CTRX and for 2 weeks with synergistic GM	Complete resolution
Our case	Nakamura et al.	57	Male	2020	Neurogenic bladder, Alcoholic cirrhosis	3 days fever and chills	MALDI-TOF–MS	Acute pyelonephritis	CTRX → add ABPC → LZD PO	Complete resolution

Vancomycin, VCM; tobramycin, TOB; flomoxef, FMOX; ampicillin, AMPC; gentamicin, GM; rifampin, RFP; PO, oral administration, Levofloxacin, LVFX; trimethoprim-sulfamethoxazole, ST; Amoxicillin, AMPC; Ceftriaxone, CTRX; Ciprofloxacin, CPFX; Cefazolin, CEZ; Cefmetazole; CMZ, cefotaxime; CTX, metronidazole; MNZ, amikacin; AMK, piperacillin/tazobactam; PIPC/TAZ, linezolid; LZD, Cefepime, CFPM; benign prostatic hyperplasia, BPH; amoxicillin-clavulanic acid, AMPC/CVA; penicillin G, PCG; azithromycin, AZM, ESRD, end-stage renal disease; DM, diabetes mellitus; HT, hypertension; cefuroxime axetil CXM-AX; chronic obstructive pulmonary disease, COPD; deep vein thrombosis, DVT

In this review, one case of death due to biliary tract infection caused by *E. hirae* was reported [12]. The mortality rate (n = 1, 3%) from *E. hirae* infection was similar to or lower than that of other *Enterococcus* spp*.* infections (23%) [23]. However, the accumulation of *E. hirae* infections warrants accurate evaluation.

Three cases of *E. hirae* infection recurred during treatment [6, 7, 21], and two of the three recurrent cases involved infective endocarditis. In a report comparing 3308 cases of infective endocarditis caused by non-*Enterococcus* spp. with 516 cases of infective endocarditis caused by *Enterococcus* spp*.* collected prospectively from 35 centers in Spain, recurrence was significantly higher in cases of infective endocarditis caused by *Enterococcus* spp. (3.5% vs. 1.7%) [28]. There were nine reported cases of *E. hirae* urinary tract infections with no recurrences or deaths.

The susceptibility of *E. hirae* to antimicrobial agents is similar to that of *E. faecalis*, which is susceptible to penicillin. Table 3 shows the antimicrobial susceptibility of *E. hirae* infections in humans. Although some reports have reported high resistance to gentamicin [29], of the 21 antimicrobial-susceptible cases in this review, only four (19%) were gentamicin-resistant, and high-level gentamicin resistance cases were not reported*.* The relatively low mortality and antimicrobial resistance suggest that *E. hirae* is more similar to *E. faecalis* than *E. faecium*. In the present case, the patient could not tolerate ampicillin due to drug allergy and was successfully treated with linezolid after confirming susceptibility. Resistance to clindamycin and gentamicin has been reported repeatedly, and the possibility of resistance should be considered when these drugs are used. The accumulation of human clinical data is warranted to generate an accurate evaluation.

**Table 3:** Summary of antimicrobial susceptibility in the previously reported human cases with *Enterococcus hirae*

Case	Sensitive	Resistance	Supplement
1	ABPC, VCM, IPM GM	N/A	No beta-lactamase activity
2	N/A	N/A	
3	N/A	N/A	
4	ABPC, VCM, TEIC, CP	CLDM, EM, RFP, TC	Low-level resistance to SM, KM, GM
5	N/A	N/A	
6	N/A	N/A	
7	N/A	N/A	
8	N/A	N/A	
9	N/A	N/A	
10	ABPC, PCG, CP, CPFX, OFLX, LVFX, TC, VCM	CLDM, NFLX	No evidence of high-level aminoglycoside resistance to GM or SM Intermediate susceptibility to EM, NTF
11	N/A	N/A	
12	N/A	N/A	
13	N/A	CLDM, Cephalosporins	
14	EM, CP, LZD, VCM	RFP	Intermediate susceptibility to ABPC, DOXY
15	ABPC, TEIC, VCM, high dose GM	OFLX, GM	
16	ABPC, TEIC, VCM, high dose GM	OFLX, GM	
17	ABPC, MFLX, VCM, TEIC, EM, RFP	CLDM, FOS	Low-level resistance to SM, KM, GM
18	ABPC, VCM, TEIC, EM, TC high-level SM and GM	N/A	
19	AMPC	Cephalosporins	
20	N/A	N/A	
21	ABPC, VCM, TEIC, LZD, TC	CPFX	No high-level resistance to GM
22	ABPC, VCM, CPFX	N/A	
23	high-level GM and KM, ABPC, LZD, CPFX, Nitrofuran, VCM	ST	
24	AMPC/CVA, PIPC/TAZ	CXM-AX, NTF	
25	VCM	N/A	
26	ABPC, ABPC/SBT, CPFX, EM, high-level GM, IPM, LVFX, LZD, NFLX, PCG, QPR/DPR, high-level SM, TEIC, TC, VCM, TGC	none	intermediate susceptibility to NTF
27	ABPC, ABPC/SBT, CPFX, EM, high-level GM, IPM, LVFX, LZD, NFLX, PCG, QPR/DPR, high-level SM, TEIC, TC, VCM, TGC	none	intermediate susceptibility to NTF
28	N/A	N/A	
29	ABPC, IPM, GM, CPFX, LVFX, VCM, TEIC, ST, LZD, TGC	N/A	
30	ABPC, AMPC, VCM	N/A	It demonstrated synergy with GM and SM
31	ABPC, VCM, high-level GM	N/A	
32	ABPC, PCG, VCM, LVFX, TEIC, LZD	none	

Ampicillin, ABPC; Vancomycin, VCM; Imipenem, IPM; Gentamicin, GM; Teicoplanin, TEIC; Chloramphenicol, CP; Clindamycin, CLDM; Erythromycin, EM; Rifampin, RFP; Streptomycin, SM; Kanamycin, KM; Penicillin G, PCG; CPFX, Ciprofloxacin; Levofloxacin, LVFX; Tetracycline; TC; Linezolid, LZD; doxycycline, DOXY; Moxifloxacin, MFLX; Amoxicillin, AMPC; Amoxicillin-clavulanic acid, AMPC/CVA; Piperacillin/tazobactam, PIPC/TAZ; Ampicillin sulbactam, ABPC/SBT; trimethoprim-sulfamethoxazole, ST; Cefuroxime axetil, CXM-AX; Norfloxacin, NFLX; Quinupristin/Dalfopristin, QPR/DPR; Fosfomycin, FOS; Tigecycline, TGC; Ofloxacin OFLX; Nitrofurantoin, NTF

Matrix assisted laser desorption ionization-time of flight mass spectrometry (MALDI-TOF MS) was developed in the 1980s and was accurate in 80–95% of bacterial isolates [13]. Species-level identifications have been obtained and have been widely used in recent years [13]. A study validated the accuracy of MALDI-TOF MS for the identification of *Enterococcus* spp. compared with the gold standard *rpoA* gene sequencing method for the identification of bacteria of environmental origin. The occurrence of *Enterococcus* spp., including *E. hirae*, in wild birds was correctly identified by MALDI-TOF MS [30]. Before the advent of MALDI-TOF–MS, *E. hirae* may have been underdiagnosed because of the limitations of the diagnostic method [3]. This review found that there has been an increase in reporting of *E. hirae* since 2015 following the advent of MALDI-TOF MS.

*Enterococcus hirae* is a newly recognized causative pathogen of urinary tract infections, especially in patients with underlying diseases. Clinical data such as risk factors, clinical manifestations, and antimicrobial susceptibility are lacking, and more cases should be accumulated following accurate identification.

In summary, the number of *E. hirae* infections reported has increased following the development of MALDI-TOF MS. Although *E. hirae* may have a low virulence, as do other *enterococci,* clinicians need to consider *E. hirae* as a causative pathogen of urinary tract infection.

## Data Availability

Not applicable.

## Abbreviations

*E.** hirae*: *Enterococcus hirae*
IV: Intravenous
MALDI-TOF MS: Matrix-assisted laser desorption ionization-time of flight mass spectrometry
WBC: White blood cell
